# Quantitative Surface-Enhanced Raman Spectroscopy: Challenges, Strategies, and Prospects

**DOI:** 10.3390/molecules31010191

**Published:** 2026-01-05

**Authors:** Zhixuan Lu, Jun Wang, Sen Yan

**Affiliations:** 1 Xiamen Key Laboratory of Optoelectronic Materials and Advanced Manufacturing, Institute of Luminescent Materials and Information Displays, College of Materials Science and Engineering, Huaqiao University, Xiamen 361021, China; 2 State Key Laboratory of Physical Chemistry of Solid Surfaces, Collaborative Innovation Center of Chemistry for Energy Materials (iChEM), College of Chemistry and Chemical Engineering, Xiamen University, Xiamen 361005, China; 20520210156366@stu.xmu.edu.cn

**Keywords:** SERS, quantitative, analysis, reproducibility, chemometrics

## Abstract

Surface-Enhanced Raman Spectroscopy (SERS) is highly attractive as an analytical technique owing to its high sensitivity, distinctive molecular specificity, and speed of analysis. It offers the potential to match the sensitivity and molecular specificity of established techniques like Gas Chromatography–Mass Spectrometry in a more affordable, faster, and portable format, providing unique solutions for challenging analytical problems such as bedside diagnostics and in-field forensic analysis. Despite these benefits, SERS currently remains a specialized technique and has not yet successfully entered the mainstream of analytical chemistry. This transition is hindered primarily by challenges in achieving robust, reliable, and especially quantitative measurements in real-world applications. Achieving quantitative SERS requires addressing core issues arising from the heterogeneous nature of enhancing substrates and the complexity of real-life samples. This perspective summarizes the fundamental challenges associated with signal variability and matrix interference. It then details modern strategies focused on standardizing performance metrics, with particular emphasis on the newly proposed SERS Performance Factor for substrate evaluation, alongside the development of advanced quantification methods (e.g., internal standardization and digital SERS) and rapid sample pretreatment protocols. Finally, emerging prospects, including the deployment of Artificial Intelligence for enhanced analysis and advancements in deep-tissue SERS sensing, are explored as critical drivers for integrating SERS into routine analytical practice.

## 1. Introduction

Surface-enhanced Raman spectroscopy (SERS) is a powerful vibrational spectroscopy technique that provides a dramatic amplification of the inherently weak Raman scattering signal from analyte molecules. This enhancement is primarily achieved when molecules are located in close proximity to nanostructured plasmonic materials, typically gold or silver, through the generation of intense localized electromagnetic fields upon laser excitation. Since its initial discovery in the 1970s [1,2], SERS has transitioned from a fundamental scientific curiosity to a well-established analytical tool, a progression greatly accelerated by parallel advances in nanotechnology and instrumental optics [1,2,3,4,5,6,7]. The principal advantages of SERS over other analytical techniques, such as chromatography or standard fluorescence assays, are its exceptional sensitivity—capable of reaching the single-molecule level [8,9]—its superb molecular specificity providing unique vibrational “fingerprints,” and its potential for rapid, non-destructive, and portable analysis [3,7,10,11].

The formidable capability of SERS for qualitative analysis is undeniable. The technique’s high specificity allows for the definitive identification of chemical species in complex mixtures, making it invaluable in scenarios such as forensic analysis of trace evidence, detection of specific pathogens, and identification of illegal adulterants in food and pharmaceuticals. However, for SERS to realize its full potential and transition into routine analytical practice, robust quantitative analysis is essential. The ability to accurately determine analyte concentration is a critical requirement in numerous fields, including therapeutic drug monitoring in clinical serum samples, quantifying environmental pollutants in water, and measuring biomarkers for disease diagnosis and staging [12,13,14]. Unfortunately, the very mechanisms that bestow SERS with its remarkable sensitivity also pose significant challenges to reliable quantification. The intense SERS signal is highly localized to nanoscale “hot spots,” making it extremely sensitive to the precise adsorption and position of analyte molecules, which leads to substantial signal heterogeneity [6,10,15]. Furthermore, factors such as the non-uniform distribution of enhancing sites, batch-to-batch variations in substrate fabrication, and matrix effects in real-world samples that alter molecular adsorption introduce significant variability. Consequently, transforming SERS from a highly sensitive and specific fingerprinting technique into a robust and reliable quantitative platform represents a pivotal, yet challenging, objective in the field. Addressing these challenges is paramount to unlocking the full scope of SERS applications and solidifying its role as a next-generation analytical methodology. This review aims to chart this journey, examining the obstacles and presenting the sophisticated strategies being developed to overcome them.

## 2. Fundamental Mechanisms of SERS Enhancement

The extraordinary signal amplification in SERS arises from two primary, often synergistic, mechanisms: electromagnetic enhancement and chemical enhancement [6,10]. A clear understanding of these mechanisms is essential for designing effective SERS substrates and interpreting quantitative results.

Electromagnetic enhancement is the dominant contributor, often accounting for signal amplifications of 10^4^–10^8^. It originates from the excitation of localized surface plasmon resonances (LSPRs) in nanostructured noble metals (typically Au or Ag). When illuminated with light at or near the plasmon resonance frequency, conduction electrons oscillate collectively, generating highly confined and intense electromagnetic fields at specific nanoscale regions known as “hot spots” [11,16]. The enhanced electric field *E*_loc_ at such hot spots amplifies both the incident laser field and the Raman-scattered field, leading to an overall Raman intensity enhancement roughly proportional to |*E*_loc_/*E*_0_|^4^. Hot spots are most pronounced in sub-10 nm gaps between nanoparticles (gap plasmons), at sharp tips, edges, or within carefully engineered nanogaps in core–shell structures. Importantly, hot spots are not exclusive to nanoparticle aggregates; they can also be generated on smooth but periodically modulated metal surfaces (e.g., grating-coupled SERS, nanohole arrays) through propagating surface plasmons or hybrid modes [17,18,19], though the field confinement may differ from gap-based systems.

Chemical enhancement, typically contributing a factor of 10^1^–10^3^, involves electronic interactions between the analyte molecule and the metal surface [10,12]. This mechanism includes resonance Raman effects (if the molecule absorbs at the excitation wavelength) and charge-transfer processes, where electrons transiently move between the metal and adsorbed molecule, altering the polarizability and thus the Raman cross-section. Chemical enhancement is highly molecule- and surface-specific, depending on molecular orientation, adsorption geometry, and the electronic structure of both the molecule and the substrate. While weaker than electromagnetic enhancement, it adds an additional layer of selectivity and can influence quantitative reproducibility through adsorption-dependent signal variations.

In practice, both mechanisms often operate simultaneously, and their relative contributions depend on the substrate morphology, excitation wavelength, and molecular identity. Acknowledging this dual mechanism framework is crucial for addressing the challenges in quantitative SERS, as both electromagnetic field heterogeneity and adsorption-dependent chemical effects contribute to signal variability.

## 3. Core Challenges Compromising SERS Quantitation

The pursuit of reliable quantification in SERS faces fundamental obstacles rooted in both the intrinsic physics of the enhancement mechanism and the practical complexities of real-world detection systems [4,20,21]. The fundamental challenge in quantitative SERS stems from the intrinsic nature of the enhancement mechanism, where the measured signal is not uniformly proportional to the bulk analyte concentration but is dominated by the local electromagnetic field enhancement (approximately |E_local_|^4^) and the number of molecules residing in these enhanced regions (N_hotspots_). This dependence creates multiple sources of irreproducibility that are inherent to most SERS systems [16,22,23]. Firstly, spatial inhomogeneity is unavoidable as plasmonic substrates—whether colloidal aggregates or solid-state chips—possess inherently heterogeneous distributions of electromagnetic “hot spots.” This results in significant point-to-point signal fluctuations for identical samples, with relative standard deviations (RSDs) of 5–15% being common even for commercial substrates. Secondly, temporal instability presents a major challenge, particularly for the widely used colloidal suspensions where the SERS signal intensity evolves dynamically with the aggregation state—initially rising as aggregates form, reaching a maximum, and subsequently decaying due to particle sedimentation. This temporal evolution makes precise control of measurement timing absolutely critical. Furthermore, batch-to-batch inconsistency remains a persistent issue, as reproducible synthesis of SERS substrates with identical plasmonic properties proves non-trivial, with variations in nanoparticle size, shape, and aggregation state between different batches potentially leading to orders-of-magnitude differences in average enhancement. These intrinsic factors collectively undermine the establishment of a reliable, predictable relationship between analyte concentration and SERS signal intensity.

**Figure 1:**
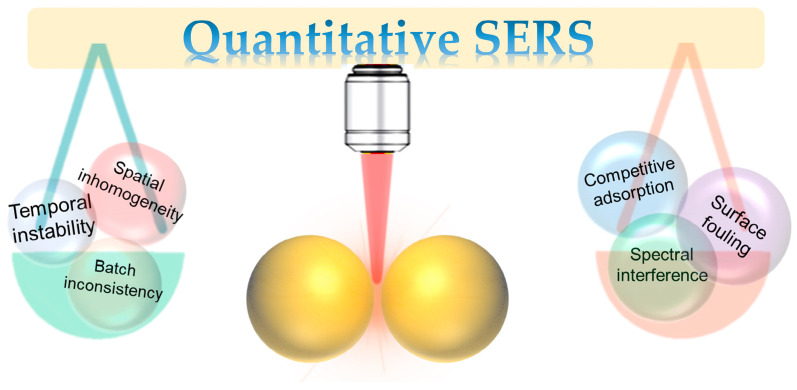
Schematic illustrating the major intrinsic and extrinsic challenges that impede reliable quantification.

Beyond these intrinsic factors, the extrinsic challenges arising from the detection system and sample matrix are equally formidable. The complexity of real samples, such as biological fluids or environmental extracts, introduces severe matrix effects [13,14]. A primary issue is competitive adsorption, where myriad non-target molecules in the sample compete for the limited adsorption sites on the SERS-active surface. This can prevent the target analyte from accessing the crucial hot spots, leading to signal suppression and a breakdown of the calibration [7,11]. Furthermore, spectral interference occurs when the SERS signals from co-adsorbed matrix components overlap with the characteristic peaks of the target analyte, complicating spectral interpretation and accurate integration of the target’s signal. Another critical challenge is surface fouling or contamination, where macromolecules like proteins form a passivating layer on the substrate. This “biofouling” can physically block hot spots, alter the local dielectric environment, and change the enhancement properties, thereby degrading analytical performance and leading to signal drift over time. These combined intrinsic and extrinsic factors create a complex web of interdependencies that must be systematically addressed to achieve robust SERS quantification (Figure 1).

## 4. Strategies for Enhancing SERS Quantitation

The transition of SERS from a qualitative fingerprinting technique to a robust quantitative analytical method requires systematic strategies that address its inherent challenges, namely the unreliable evaluation of enhancement and the significant signal variance. This section outlines a cohesive pathway for enhancing SERS quantification, beginning with the establishment of a reliable performance standard, moving to innovative quantification methodologies, and concluding with practical considerations for analyzing complex, real-world samples.

Establishing a standard: From EF and AEF to the SERS Performance Factor (SPF)

**Figure 2:**
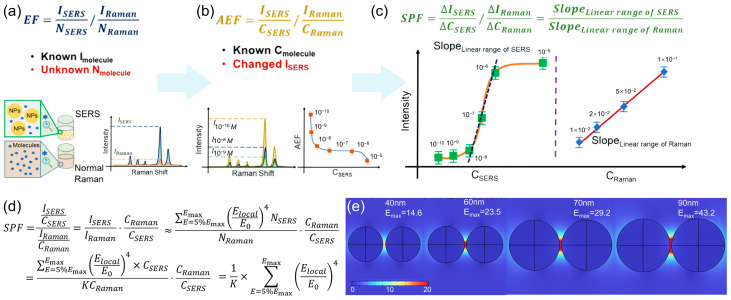
Schematic definition of EF (**a**), AEF (**b**), and SPF (**c**). (**d**) The SPF expression with the theoretical expression of I_Raman_ and I_SERS_. (**e**) The electromagnetic filed distribution simulation results of different diameter Au NPs. Reproduced with permission from refs. [21,24].

A fundamental barrier in comparing SERS substrates and methods has been the lack of a consistent and practical standard for evaluating enhancement [24]. The traditional Enhancement Factor (EF, Figure 2a), defined as EF = (I_SERS_/N_SERS_)/(I_Raman_/N_Raman_), conceptually captures the essence of SERS enhancement per molecule [16,23]. However, its practical application is severely hampered by the extreme difficulty in accurately determining N_SERS_, the number of molecules contributing to the SERS signal within the plasmonic hot spots. Estimates of N_SERS_ involve challenging parameters like hot-spot area (A), molecular footprint (σ), and surface coverage (θ), leading to EF values that can vary by orders of magnitude even for identical substrates. To circumvent this, the Analytical Enhancement Factor (AEF, Figure 2b) was introduced: AEF = (I_SERS_/C_SERS_)/(I_Raman_/C_Raman_). This parameter is more experimentally accessible as it uses solution concentrations (C_SERS_ and C_Raman_) instead of molecular numbers. While practical, AEF is highly dependent on the specific concentrations used and does not account for the analyte’s transfer from the bulk solution to the surface, making it susceptible to variations in experimental conditions and difficult to model theoretically. Addressing these limitations, the SERS Performance Factor (SPF, Figure 2c) has been proposed as a more robust and convenient parameter [24]. It shares the same mathematical form as AEF, SPF = (ΔI_SERS_/ΔC_SERS_)/(ΔI_Raman_/ΔC_Raman_), but its derivation and application are fundamentally different. SPF is obtained by taking the ratio of the slopes of the linear regions in concentration-dependent SERS and normal Raman calibration curves.

This approach offers critical advantages: Concentration independence: By leveraging the linear relationship between signal and concentration (under sub-monolayer coverage), SPF becomes a constant, intrinsic property of the substrate-probe system, independent of the specific concentration points chosen. Theoretical significance: SPF quantitatively describes the overall contribution to SERS performance, integrating both the electromagnetic enhancement of the substrate and the interfacial interaction (adsorption efficiency) between the probe and substrate. Its theoretical derivation shows a direct correlation with the sum of the enhanced electromagnetic field ((E_local_/E_0_)^4^) in the hot spots (Figure 2d). Experimental validation: Studies on size-tunable Au nanoparticles (both colloidal and solid) have demonstrated that the trend in SPF aligns perfectly with the trend in substrate sensitivity (as indicated by the lowest detectable concentration, LDC). Furthermore, FDTD simulations of Au dimers of varying sizes show a consistent size-dependent trend between the theoretical SPF (calculated from simulated EM fields) and the experimentally obtained SPF, confirming its reliability as an evaluation metric (Figure 2e). The adoption of SPF provides a consolidated and reliable protocol for cross-laboratory evaluation of SERS substrates, forming a foundational step towards standardizing SERS technique.

Advanced quantification methodologies

The establishment of a reliable standard like SPF provides a crucial foundation for comparing SERS substrates. However, achieving accurate and precise quantification in practice requires innovative methodologies designed to combat the intrinsic sources of signal variance in SERS experiments. These methods move beyond simply measuring intensity and instead incorporate built-in correction, statistical analysis, and intelligent data processing to deliver reliable results.

**Figure 3:**
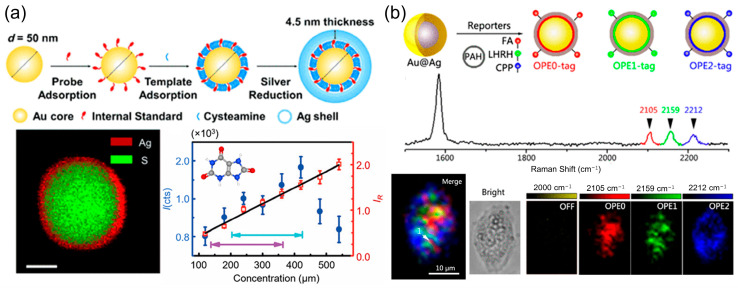
(**a**) Synthetic scheme for the Au@CA + MPy@Ag NPs (CMS NPs). TEM-DES image showing the composition of a single particle. Calibration plots for detection of uric acid. The normal human concentration ranges are marked with turquoise (male) and blue (female) arrows. Reproduced with permission from ref. [25]. (**b**) Three-color SERS imaging of HeLa cells using SERS nanotags of an alkyne SERS palette; the SERS nanotags were modified with OPE0 (red), OPE1 (green), and OPE2 (blue). Reproduced with permission from ref. [30].

A cornerstone strategy for improving quantitative precision is the use of internal standardization. This approach recognizes that many sources of fluctuation—such as variations in laser power, focal position, or the local density of plasmonic hot spots—affect all molecules in the probe volume similarly [4]. By normalizing the target analyte’s signal (I) to that of a reference molecule (I_0_), these variations can be effectively canceled out. The simplest implementation involves pre-adsorbing a robust Raman reporter (e.g., a thiolate compound) onto the SERS substrate prior to analyte exposure. While this effectively corrects for physical instabilities, it cannot account for chemical competition, where components of a complex sample matrix alter the adsorption efficiency of the target itself. Ren and colleagues engineered a sophisticated core/shell nanoparticle system, featuring a gold nanosphere core encapsulated by a hybrid layer of cysteamine and 4-mercaptopyridine (4-MPy), all coated within a silver shell (Figure 3a). This architecture strategically isolates the 4-MPy internal standard within the particle, freeing the outer surface for unhindered analyte interaction [25]. When applied to uric acid detection across physiologically relevant levels, the raw SERS signal exhibited considerable non-linearity. However, upon normalizing the uric acid signal against the encapsulated 4-MPy signal, the calibration curve transformed into a highly linear relationship, demonstrating the efficacy of this built-in correction method. This method corrects for both physical and chemical variances, and has been demonstrated to maintain calibration accuracy across different instruments and over periods of several years, representing a gold standard for demanding applications.

Gap-enhanced Raman tags (GERTs) represent a significant advancement in SERS-based quantification, offering ultrahigh brightness and exceptional signal stability due to their unique core–shell structure with built-in Raman reporters embedded in sub-nanometer interior gaps [26,27,28]. The electromagnetic enhancement within these gaps is maximized when the gap size is optimized, typically around 1.2–1.6 nm, leading to highly reproducible and intense SERS signals. This structural stability allows GERTs to maintain consistent SERS responses even in aggregated states, making them particularly suitable for quantitative Raman analysis. A key development in this area has been the introduction of orthogonal-GERTs by Shen and colleagues [29,30], who embedded alkyne- and deuterium-based Raman reporters within the nanogaps. These reporters exhibit distinct vibrational peaks in the biologically silent region (1800–2800 cm^−1^), minimizing interference from endogenous biomolecules and enabling accurate quantification in complex media (Figure 3b). Such designs not only improve photostability and reduce background interference but also facilitate multiplexed detection by allowing simultaneous encoding of multiple Raman signatures. The reliable and reproducible signal output of GERTs, especially when combined with resonance Raman effects or tailored core–shell morphologies (e.g., petal-like or multishell structures), provides a robust platform for quantitative biomedical applications, including biosensing, single-cell analysis, and in vivo imaging [30].

**Figure 4:**
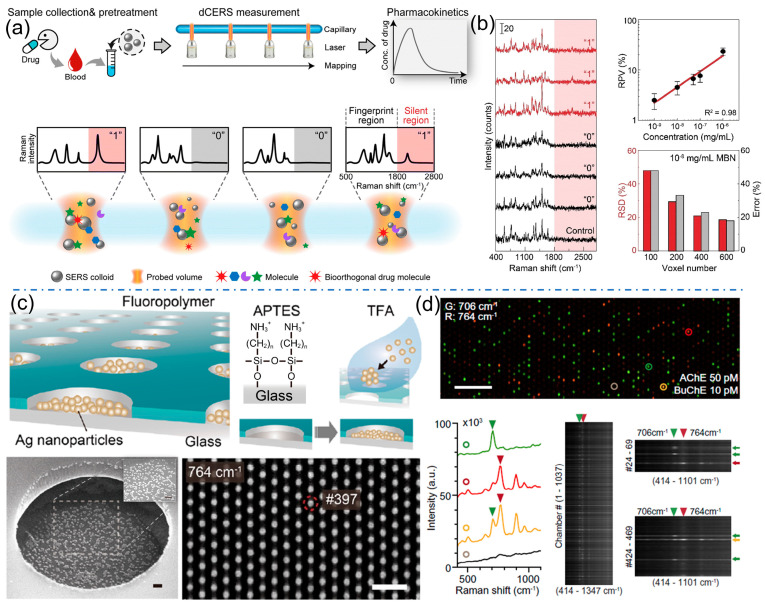
(**a**) The concept of digital colloid enhanced Raman spectroscopy by single-molecule counting. (**b**) Typical positive (“1”) and negative (“0”) spectra of MBN and a typical spectrum of the control sample. Calibration curve of MBN by dCERS, and the voxel number-dependent quantification accuracy at 10^−8^ mg per mL MBN. Reproduced with permission from ref. [33]. (**c**) Schematic of the SERS device, the bottom images are SEM and SERS image at 764 cm^−1^ of a 100 μM thiocholine solution (**d**). Dual-color SERS image at 706 cm^−1^ (green) and 764 cm^−1^ (red) of a mixture containing 50 pM AChE, 10 pM BuChE, 1.5 mM MATP+, and 4 mM butyrylthiocholine (Scale bar: 50 μm), with SERS spectra of the chambers indicated by green, red, yellow, and brown circles. Right bottom SERS spectra extracted from each chamber in Dual-color SERS image. Reproduced with permission from ref. [35].

While internal standards tackle variance at conventional concentrations, a paradigm shift in methodology is required at the ultra-low, single-molecule level. Here, the random adsorption of molecules into highly non-uniform plasmonic hot spots leads to enormous, unpredictable fluctuations in SERS intensity, rendering traditional intensity-based calibration useless [31,32]. Digital SERS overcomes this by redefining the measurand from “signal intensity” to “binary event count.” In this approach, a large sample area is systematically mapped, and each spectrum is classified simply as containing a SERS event (“on”) or not (“off”). The analyte concentration is then derived statistically from the fraction of active hot spots, typically following a Poisson distribution. Ye group introduced a digital colloid-enhanced Raman spectroscopy (dCERS) platform (Figure 4a). This method leverages a colloidal system where both plasmonic hotspots and target molecules are randomly and uniformly distributed at the probe-volume level [33,34]. Under these conditions, the binding of analyte molecules to hotspots follows a Poisson distribution, enabling digital single-molecule counting. The reproducibility and accuracy of this assay at ultralow concentrations are thus governed by the statistical power of accumulated single-molecule event counts, offering a robust solution that meets practical demands for both precision and analysis time. This digital counting process effectively linearizes the response at attomole to femtomolar concentrations, pushing the limits of quantification to unprecedented lows and opening up new possibilities for trace analysis in fields like clinical diagnostics and environmental monitoring (Figure 4b). The pioneering work of Ando et al. has recently introduced the innovative concept of digital SERS [35]. This platform leverages plasmonic microchamber arrays to compartmentalize and detect single-enzyme reactions (Figure 4c). By converting the generation of a SERS-active product into a binary digital readout, the method achieves femtomolar sensitivity and true multiplexing capability based on distinct molecular fingerprints, thereby overcoming the selectivity limitations of traditional fluorescence-based digital assays and opening a new avenue for the precise quantification of multiple low-abundance biomarkers in complex samples (Figure 4d).

For the most complex scenarios involving multi-analyte mixtures or high-dimensional spectral data, univariate analysis of single peaks becomes inadequate. This is where chemometrics and artificial intelligence (AI) step in. Multivariate techniques like Partial Least Squares (PLS) regression can model the entire spectral shape, deconvoluting overlapping peaks and accounting for subtle matrix-induced baseline shifts [36]. Going further, machine learning algorithms, such as Support Vector Machines (SVMs) and Artificial Neural Networks (ANNs), can be trained to recognize complex, non-linear patterns in SERS data. For instance, AI-driven “SERS tasters” have been developed to simultaneously quantify multiple flavor molecules in a complex matrix like wine by learning the subtle spectral perturbations induced by non-covalent interactions between analytes and a reporter layer [37]. These powerful data processing techniques transform the SERS spectrum from a simple fingerprint into a rich, high-dimensional data source, enabling accurate quantification even when traditional methods fail.

The evolution of quantitative SERS is marked by a transition from relying on raw signal intensity to employing sophisticated methodological frameworks. The sequential or combined application of internal standardization, digital SERS, and AI-assisted chemometrics provides a powerful toolkit to neutralize variance, conquer the single-molecule regime, and decipher complex mixtures, thereby solidifying the role of SERS as a versatile and reliable quantitative analytical technique.

Navigating real-world complexity: Sample pretreatment and substrate design

**Figure 5:**
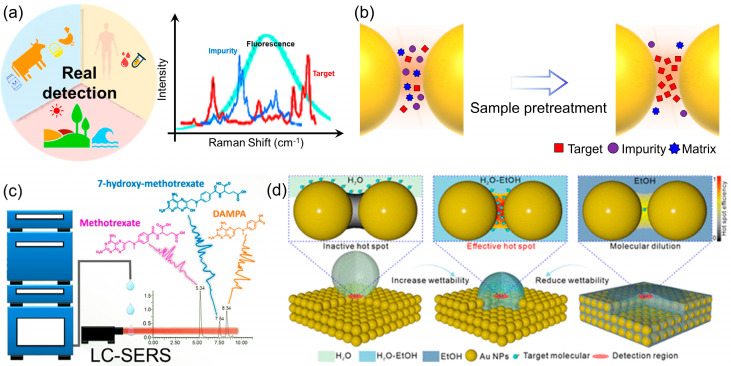
(**a**) Schematic illustration of SERS detection to real samples. (**b**) Diagram of actual sample pretreatment, more samples can be incorporated into the hotspot. (**c**) Quantitative Online Liquid Chromatography–Surface-Enhanced Raman Scattering of methotrexate. Reproduced with permission from ref. [41]. (**d**) The EtOH–H_2_O mixture reduces the surface tension of the solvent, increases the solvent wettability, and significantly improves the efficiency of target molecules entering the hot spots. Target molecules are able to enter the nanogap, effectively activating the hot spot. Reproduced with permission from ref. [45].

The ultimate validation of any quantitative analytical technique lies in its performance against complex, real-world samples [38,39]. For SERS, this transition from idealized laboratory solutions to intricate matrices such as biological fluids, food products, or environmental water represents a significant hurdle (Figure 5a). The primary challenge stems from matrix effects, where interfering compounds—proteins, salts, organic matter, and other constituents—can foul the substrate surface, compete with the target analyte for adsorption sites, quench the plasmonic enhancement, or generate a confounding spectroscopic background. To overcome these barriers and unlock the practical potential of SERS, a dual strategy encompassing robust sample preparation and intelligent substrate engineering has become indispensable (Figure 5b).

In practice, some degree of sample pretreatment is often a necessary first step to simplify the matrix and mitigate its adverse effects. Traditional methods such as liquid–liquid extraction, solid-phase extraction, and filtration remain highly effective for purifying and pre-concentrating analytes from complex backgrounds [40]. For instance, extracting a pesticide from a fruit homogenate or a drug metabolite from serum can dramatically reduce interference and improve detection reliability. A more sophisticated approach involves hyphenating SERS with a separation technique, most notably liquid chromatography (LC-SERS). Royston Goodacre’s group demonstrated the power of online LC-SERS for managing complex biological matrices by quantitatively monitoring methotrexate and its metabolites in human urine (Figure 5c). They developed a reversed-phase LC system with gradient elution coupled to a post-column SERS detection setup using silver colloid [41]. This approach effectively separated the analytes, circumventing the spectral overlap typical in direct SERS of urine, and provided molecularly specific identification and quantification with detection limits in the low micromolar range, successfully validating the method with patient samples. This powerful synergy effectively decouples the identification and quantification of the target from the matrix, offering a robust solution for multi-analyte determination in challenging samples.

While pretreatment is effective, it adds steps, time, and complexity, potentially undermining SERS’s advantage as a rapid technique. This limitation has catalyzed the development of “smart” multifunctional SERS substrates designed to actively manage matrix complexity in situ. The core principle is to engineer substrates with built-in functionalities that go beyond mere signal enhancement [42,43]. A key strategy involves imparting molecular selectivity by functionalizing the plasmonic surface with capture agents such as antibodies, aptamers, or molecularly imprinted polymers [43,44]. These receptors act as synthetic antibodies, selectively binding the target analyte while repelling interferents, thereby ensuring that the enhanced field is exclusively occupied by the molecule of interest. Beyond selectivity, combating non-specific adsorption—a major issue in biofluids—is critical. This has been addressed by designing substrates with integrated anti-fouling properties. Coating the nanostructures with bio-inert materials like zwitterionic polymers creates a hydration layer that effectively resists the adhesion of proteins and other biomacromolecules. Such substrates maintain their functionality and sensitivity even when directly immersed in crude plasma or serum, enabling direct quantitative analysis with minimal sample handling. Yang et al. addressed a fundamental yet often overlooked bottleneck in quantitative SERS: the inefficient delivery of analyte molecules into plasmonic hot spots (Figure 5d). They identified that airborne hydrocarbon adsorption renders nanostructures hydrophobic, severely hindering aqueous sample infiltration [45]. Their innovative solution employs a tunable ethanol-water binary solvent to precisely control interfacial wettability and surface tension, thereby actively guiding diverse molecules into the nanogaps. This simple yet universal strategy enhances detection limits by 2–3 orders of magnitude and significantly improves signal reproducibility, providing a crucial practical advancement for reliable SERS quantification. Furthermore, the integration of internal standards directly into the substrate architecture, as discussed earlier, provides an internal calibration that corrects for signal fluctuations, making the quantification more reliable in variable matrix conditions.

Looking forward, the convergence of these functionalities—selective capture, anti-fouling protection, and built-in calibration—into a single, integrated “lab-on-a-particle” or “lab-on-a-chip” platform represents the cutting edge of quantitative SERS [46,47]. These multifunctional sensors aim to handle the sample matrix autonomously, purifying, enriching, and quantifying the target analyte in a single step. By shifting the burden of complexity from the user and the protocol to the substrate itself, these advanced material designs are poised to bridge the gap between demonstrated laboratory potential and the demanding requirements of routine, real-world quantitative analysis.

## 5. Prospects: AI, Deep Sensing, and Future Directions

The evolution of surface-enhanced Raman scattering from a powerful spectroscopic phenomenon into a robust quantitative analytical technique is poised to enter a transformative phase. Future advancements will be driven by a deeper integration with cutting-edge technologies from computational science, materials engineering, and biophysics, pushing the boundaries beyond conventional sensitivity and reproducibility metrics. The trajectory points towards the development of intelligent, minimally invasive, and information-rich sensing platforms capable of addressing complex real-world challenges.

The application of machine learning (ML) and deep learning in SERS is set to revolutionize data processing and quantitative analysis [48]. Traditional SERS quantification is often hampered by spectral noise, background interference, and substrate heterogeneity. AI algorithms can automate and optimize critical steps such as spectral denoising, baseline correction, and peak fitting, significantly improving signal clarity and reproducibility [40,49,50]. For instance, unsupervised learning models can perform high-throughput preprocessing, enabling real-time data handling in complex environments such as biological fluids or environmental samples. Moreover, interpretable ML models can identify subtle spectral variations indicative of specific analytes or intermediate species, facilitating more accurate concentration predictions and mechanistic insights. The use of graph neural networks (GNNs) and other deep learning architectures also allows for the prediction of spectral features from molecular structures, bridging the gap between experimental and theoretical spectra [51]. This AI-driven approach not only enhances the reliability of quantitative SERS but also paves the way for adaptive and transferable models across different instruments and experimental conditions.

The next frontier for SERS lies in extending its reach to in vivo and non-invasive applications. Deep-tissue Raman detection techniques, such as spatially offset Raman spectroscopy and transmission Raman spectroscopy, have already demonstrated penetration depths of several millimeters to centimeters, opening avenues for medical diagnostics and monitoring. Coupled with SERS, these methods can enable the detection of disease biomarkers or drug molecules deep within tissues without invasive procedures. Concurrently, the development of flexible, biocompatible, and wearable SERS substrates is gaining momentum. Such devices can be integrated into patches, textiles, or implantable sensors for continuous molecular monitoring—such as tracking metabolites, hormones, or toxins in sweat, interstitial fluid, or blood. Advances in nanofabrication and material science will be crucial to producing robust, reproducible, and sensitive SERS platforms that function reliably under real-world conditions. These wearable systems could revolutionize personalized medicine, enabling real-time health assessment and early disease detection.

Finally, one of the most exciting prospects for SERS is its ability to not only detect but also resolve molecular structures and conformational changes [52]. The intrinsic fingerprinting nature of Raman spectra provides detailed vibrational information, which, when combined with advanced computational models, can reveal atomic-scale structural details. Recent progress in hybrid quantum-classical simulations, ab initio molecular dynamics (AIMD), and machine learning protocols (e.g., SPARC) has enabled the reconstruction of 3D protein structures and the analysis of adsorption configurations with near-atomic precision [53]. By correlating spectral features with local electric field distributions and molecular orientations, researchers can probe microenvironments at plasmonic hotspots, gaining insights into reaction mechanisms, protein folding, and interfacial phenomena. This capability is particularly valuable in structural biology, catalysis, and materials science, where understanding the relationship between structure and function is paramount. Future efforts will focus on developing real-time, on-the-fly models that integrate experimental SERS data with dynamic simulations, offering a comprehensive tool for studying complex systems such as folding proteins or evolving catalytic surfaces.

The convergence of AI, advanced sensor design, and high-fidelity spectral simulation is poised to unlock new dimensions in SERS applications [54,55]. AI-enhanced quantification will bring unprecedented accuracy and speed to molecular detection, while deep-tissue and wearable SERS sensors will expand the technique’s utility in clinical and environmental monitoring [56]. Meanwhile, advances in structural interpretation will transform SERS from a detection tool into a powerful method for molecular-level insight [57,58]. Together, these directions will ensure that SERS continues to be at the forefront of analytical science, offering both fundamental understanding and practical solutions across chemistry, biology, and medicine.

## 6. Conclusions

The path to mainstream quantitative SERS is being paved by a multi-faceted approach. The journey involves a critical understanding and control of the three core components: substrates, instrumentation, and data processing. The routine adoption of robust internal standardization, particularly using isotopologues, and the emergence of digital SERS for ultra-trace analysis are key methodological advances. Furthermore, the standardization of substrate performance through metrics like the SERS Performance Factor (SPF) is crucial for comparing and validating technologies. Finally, the leveraging of AI for intelligent data analysis and the development of advanced sensing platforms like SESORS and wearable sensors are breaking down the final barriers to application. By continuing to converge advancements in nanotechnology, instrumentation, and data science, SERS is poised to fully realize its potential as a transformative, routine quantitative tool across the chemical, biological, and medical sciences.

## Data Availability

No new data were created or analyzed in this study. Data sharing is not applicable to this article.

## Abbreviations

The following abbreviations are used in this manuscript:

SERS	Surface-Enhanced Raman Spectroscopy
RSDs	Relative standard deviations
SPF	SERS Performance Factor
EF	Enhancement Factor
AEF	Analytical Enhancement Factor
LDC	Lowest detectable concentration
FDTD	Finite-difference time domain
4MPy	4-mercaptopyridine
GERTs	Gap-enhanced Raman tags
dCERS	Digital colloid-enhanced Raman spectroscopy
AI	artificial intelligence
PLS	Partial Least Squares
SVMs	Support Vector Machines
ANNs	Artificial Neural Networks
LC	Liquid chromatography
ML	Machine learning
GNNs	Graph neural networks
AIMD	ab initio molecular dynamics
SPARC	Separable PD-based algorithm with rotational coarse-grained model
